# Employees and the National Insurance Act

**Published:** 1912-06-08

**Authors:** 


### NATIONAL INSURANCE ACT DEPARTMENT.

### EMPLOYEES AND THE NATIONAL INSURANCE ACT.

### XIV.—The Domestic Staffs in Hospitals.

It will, doubtless, be remembered that during the progress of the National Insurance Bill through Parliament a very strong protest was made against the compulsory insurance of domestic servants. Demonstrations were made at the Royal Albert Hall and at many other places throughout the country. Deputations waited upon the Chancellor of the Exchequer. In fact, every effort was made that could be made by a large number of mistresses and servants against the so-called servant tax. The demonstrations failed to achieve their full objective, but certain amendments of the original scheme resulted, and these were in due course incorporated in the Act. Domestic servants, whether single or married, will in ordinary cases be insured under the Act. This will be so whether their employers are private mistresses or institutions, such as hospitals. All grades of servants will come within the description of employed persons, within the meaning of the Act, and it is only in rare cases that the provisions allowing exemption will operate. It is probable that during the last few weeks most of the servants in large establishments have had the matter brought to their notice, either by their employers or by canvassers on behalf of societies that are becoming approved for the administration of the benefits of the Act. It is only within the past few weeks that there has been any active canvassing, on a large scale, by the societies, but now that it has begun things are moving apace. Where before there was apathy, now there is a desire for information; and where only a short time ago there was active opposition there has now appeared, as a direct result of the personal solicitation for membership of a particular society by a canvasser who is furnished with exact information as to the benefits to be derived, a keen desire to obtain the greatest benefits that are available. Insurance against sickness is an almost entirely new problem so far as women are concerned. The actuaries who made the calculations of the contributions required to provide the benefits had to report that there are no tables available upon which any reliance can be placed as to the rates of sickness of females. If actuarial experts know little or nothing about the problem, it is little wonder that some, or even most, of the domestic servants who find that they have to be insured should find difficulty in understanding how they are affected by the scheme that has been enacted for their benefit in common with the general body of employed persons.

### Official Leaflets.

In order that the matter may be more readily understood the National Health Insurance Commission have issued, during the past month, two official explanatory leaflets, which, for their own advantage, all domestic servants would do well to obtain and read carefully. The leaflets referred to are numbered 16 and 17, and bear the titles "Domestic Servants" and "Girls under Twenty-one in Domestic Service" respectively. They may be obtained free of charge, on application being made to the Secretary, National Health Insurance Commission (England), Buckingham Gate, London, S.W. There are two other leaflets in the same series numbers 7 and 11*a* respectively, which deal more generally with the insurance of women. These also may be procured in the same manner, and will repay careful attention. It may be mentioned that the leaflets are written in popular language, and they do not enter into the more complicated provisions which are quite necessary to the expert and the administrator, but only confusing to those not possessing special knowledge of insurance matters. There is, however, an important matter on which they are silent. Being official publications the leaflets cannot give any indication of the matters that should be considered before choosing a particular society as the administrator of the benefits. Any such indications, even if stated in the most general terms, might be taken as a recommendation of a particular society, or class of society, and in this matter, apart from the fact that only those societies which obtain approval are permitted to administrate the benefits, the Commissioners must of necessity be absolutely impartial.

### The Unenviable Position of the Deposit Contributor.

The society that is most suitable to the requirements of one servant is not necessarily the most suitable for another. The choice must depend on circumstances, and of these each individual must decide for herself. We are, however, in a position to indicate some considerations that should be borne in mind before making a final decision. In the first place we can say emphatically that unless a servant becomes a member of an approved society she will not obtain the full benefits of the Act. The alternative course to becoming a member of an approved society is to become a deposit contributor. There is no other alternative. A deposit contributor is one who, by failing to become a member of a society, is compelled under the provisions of the Act to obtain a card from the Post Office, on which the stamps, representing the contributions, will be stuck, and which will at the end of each quarter have to be handed back to the Post Office, so that the contributions may be placed to the credit of the special deposit account that will be opened with the local Insurance Committee. At the commencement of each year a sum will be charged to the account to cover the cost of the medical and sanatorium benefits and the cost of administrating the benefits, and such sum as may remain is then available, together with the State grant of 2d. for every 8d. expended on benefits, so far as they jointly allow, and no further, towards the provision of benefits payable during sickness. Such an arrangement lacks all the essentials of insurance, and it is to be hoped that no servant will voluntarily allow herself to become a deposit contributor. This much, at least, is made clear in the official leaflets, but no further guidance in the choice of a particular society is given.

### Points to be Considered in Choosing a Society.

In dealing with the insurance of trained hospital nurses we were able to advise, without reserve, the selection of the Nurses' Insurance Society, because this society is being formed specially and exclusively for the benefit of that particular class of employed contributors. So far as we are aware there is no society that can claim the same consideration from domestic servants as a body, and for the moment, at least, we do not intend to particularise any individual society. It may at first sight appear that the choice is immaterial, because the contributions and benefits are the same whatever society may be chosen. This may be so at the outset, but in the course of time the initial choice will, without doubt, have a considerable effect on the benefits obtainable. At the end of three years the affairs of the societies will be actuarially investigated, that is to say, a valuation will be made of the liabilities and assets, which is a similar operation, on scientific lines, to the stock-taking of a commercial trader. The result of the valuation will be to disclose either a profit or a loss on the working of the society in the intervening period. If a profit is shown the members will share in that profit in the form of increased benefits; whilst if a loss is disclosed the future benefits will be reduced. One of the prime elements in working for a profit is effective administration, and another is economical management. When a society is in a position, through having an existing organisation, to combine both these elements it stands a very fair chance from the outset of being able to provide additional benefits as the result not only of the first, but also of subsequent valuations.

### Transfers between Societies.

Another point to consider is in relation to the removal from the district in which the society operates. Provision is made in the Act for transfers from one society to another, and if a transfer is satisfactorily arranged the insured person does not suffer. There is nothing, however, in the Act to compel a society to accept an applicant, so that it is readily conceivable that a servant who elects to join a small local society, which suits her purpose admirably whilst she remains in the same situation or the same district, may find on her removal to another district that she is unable, it may be through ill-health, to obtain admission to another society, in which case she will have no option but to become a deposit contributor, with the results we have already indicated. There is on this account a very strong reason why servants should choose a society whose operations extend throughout the United Kingdom, so that removal will not necessitate transfer from one society to another, but only the notification of the change of address. Sentiment and personal acquaintance with the organisation which is forming the approved society will, of course, enter into the question. Personal confidence in the canvasser and in the society he represents will go a long way in deciding the choice that is to be made.

If, however, any of our readers to whom we have particularly addressed these remarks are in any doubt as to the most suitable society for their particular purpose, we shall be pleased to consider the circumstances of their case in greater detail in our Bureau of Information, if they will write us, enclosing with the letter the coupon that will be found on the inside of the back cover of this number. The matter of choice is of some urgence, as the Act comes into operation in about five weeks' time.

### Answers to Correspondents.

### MATERNITY BENEFIT TO PATIENTS IN AN INSTITUTION.

"Superintendent" writes: "Can the maternity benefit ever be paid to an institution? If a woman having no dependants wishes to be confined in a hospital, can she make an agreement to pay the hospital any part of the maternity benefit?"

*** The disposition of maternity benefit in the circumstances mentioned is regulated by Section 12 of the Act. When a woman is confined in a hospital and maternity benefit only is payable, the sum must be applied for the relief or maintenance of her dependants, if any. If she has no dependants and is a member of an approved society which has entered into an agreement with the hospital for the treatment of its members, the benefit may be paid, in whole or in part, according to such agreement, towards her maintenance whilst in hospital. When both the husband and wife are insured persons, the woman would become entitled to sickness benefit, in addition to the maternity benefit payable to the husband on her confinement, were it not for the fact that she is an inmate of a hospital. As the confinement is in hospital, the sickness benefit will be ear-marked for the woman's dependants, but the maternity benefit will be forfeited to the society, which may, however, give the whole or a part of it to the institution in which she is confined, provided an agreement has been entered into, as previously indicated. It must also be noted that the insured person is debarred from making any binding agreement to charge any benefit receivable under the Act (Section 111). This does not prevent a woman making a payment to the hospital voluntarily, but any agreement to charge the benefit in consideration of the treatment afforded is void.

### A POINT FOR HOSPITAL MANAGERS.

On page 260 we publish a letter from a Hospital Secretary, raising a point as to the medical attendance of indoor hospital employees, on which he invites the opinions of other hospital managers.

NOTE:—Questions and Correspondence on all doubtful points are invited, and should be addressed to the Insurance Editor, The Hospital, 28 Southampton Street, Strand, W.C.

